# The effects of a smartphone game training intervention on executive functions in youth soccer players: a randomized controlled study

**DOI:** 10.3389/fspor.2023.1170738

**Published:** 2023-08-03

**Authors:** Florian Heilmann, Damiano Formenti, Athos Trecroci, Franziska Lautenbach

**Affiliations:** ^1^ Movement Science Lab, Institute for Sport Science, Martin-Luther University Halle-Wittenberg, Halle (Saale), Germany; ^2^ Department of Biotechnology and Life Sciences, University of Insubria, Varese, Italy; ^3^ Department of Biomedical Sciences for Health, Faculty of Medicine and Surgery, University of Milan, Milan, Italy; ^4^ Department of Sport Psychology, Institute for Sport Science, Humboldt-Universität zu Berlin, Berlin, Germany

**Keywords:** executive functions, cognitive training, soccer, video game, RCT, mobile applications

## Abstract

Cognitive training primarily aims to improve executive functions (EFs). It has become a popular research topic, as previous studies have provided preliminary evidence that EFs relate to sports performance. However, whether a domain-generic cognitive training intervention can improve EFs in high-performance athletes is still unclear. The present randomized controlled study aimed to examine the effects of an eight-week (5 min/day, 5 days/week) smartphone-based domain-generic cognitive training intervention (i.e., the smartphone game “Fruit Ninja”) on EFs in youth soccer athletes (*N* = 33; intervention: *n* = 15, passive control: *n* = 18; German youth soccer academy). We assessed working memory (3-back task), inhibition (Flanker & Go/NoGo task), and cognitive flexibility (number-letter task) in a pre-post design with computerized tasks. The results showed no significant time x group differences attributable to the cognitive training between the intervention group and the control group, except for a response time variable of the Go/NoGo task. These preliminary results do not suggest an application of CT as a smartphone-based game to improve EFs performance in soccer players. However, more research is needed to establish the efficacy of domain-specific interventions in high-level team sport athletes.

## 1. Introduction

Cognitive training, especially the improvement of executive functions (EFs), has been a growing research topic in recent years (1). EFs, which refer to inhibitory control, working memory, and cognitive flexibility (e.g., see 2), enable us to think before acting, inhibit non-relevant information, resist temptations or impulsive reactions, and flexibly adapt to changed affordances as well as requirements in a varying environment (3). Moreover, EFs are related to intellectual achievements, physical and mental health, wealth, and overall quality of life (2, 4). Additionally, in the context of sports, recent studies have shown that EFs can predict sports performance (5–7), sports participation (for review, see 8) and physical activity (for reviews, see (9–12). Particularly in soccer, the relevance of cognition has been demonstrated (13–15). Overall, these notions demonstrate the importance of EFs in various aspects of daily life (2) and sports performance (e.g. 6), and thereby, suggest the potential lying in the development and improvement of EFs.

According to the cognitive skill transfer hypothesis (16), cognitive skills are either transferred only in the same context to similar tasks (near transfer) or could be generalized and transferred to far contexts (broad transfer or far transfer). Some studies postulate that a far transfer is relevant in predicting real-world performance (e.g., sport-specific context; 17). It can be argued that this far transfer may be related to athletes' expertise, that in turn might differently affect EFs. In other words, there is evidence that athletes with higher expertise in their sport tend to show higher EFs performance with respect to their lower expertise peers (8). In addition, Romeas et al. (18) focused on the far transfer of the developed domain-generic cognitive skills to the sporting domain (sport-specific domain). They found that a cognitive training (CT) task (i.e., Neurotracker software; 19) improved passing decision-making accuracy in soccer players. In this way, the authors showed the transferability from a domain-generic CT task (i.e., a 3-dimensional multiple object tracking task) in a laboratory situation to a sport-specific performance task (i.e., passing accuracy) as a far transfer. However, it remains unclear whether domain-generic CT could improve EFs that are relevant for sports performance (i.e., near transfer; e.g., 6) in high-level athletes.

Many studies have reported improvements in EFs using domain-generic commercial cognitive training tools (i.e., Cognifit, Cogmed, Lumosity, Neurotracker; for review, see (1). This means that EFs are generally trained using tasks that are also general. For example (20), showed that a domain-generic CT (i.e., “Nintendo Brain Age”) leads to improved working memory (i.e., backward digit span task). However, despite the evidence of the positive effects of different CT interventions or training devices (i.e., 21, 22), the debate about the effectiveness of CT on children (23), adults (24, 25), and older adults (21, 26, 27) is still ongoing. Brain training apps have mostly failed to demonstrate cognition benefits that effects are largely domain-specific and do not translate to the real world (28, 29). More importantly, in the sporting context, a gap exists in the knowledge about the effectiveness of domain-generic CT on high-performance athletes. Considering the importance of perceptual-cognitive skills in team sports (30, 31), investigating the potential positive effect of domain-generic CT on EFs in high-level athletes might be useful for identifying strategies to improve the cognitive domain related to sports performance (13, 15). Thus, the current randomized, controlled study aims to investigate the effects of a low-cost, smartphone-device CT intervention on EFs in young, high-level soccer players.

The transfer of cognitive skills can be expanded on the transferability of skills learned or trained with domain generic CT to a sports context (see 18). However, there is a lack of knowledge about whether improving EFs by CT tasks could impact domain-generic EFs that are relevant for sports performance (e.g., inhibition; see 6). Thus, the question of whether domain-generic CT can improve EFs (basal cognitive functions) in athletes needs to be answered, especially because athletes are a specific population, particularly when considering EF performance (e.g., 8).

In detail, studies have found that athletes have superior EFs compared to non-athletes (for review, see, 8). Various scientific working groups suggest that EF performance results from affordances in a particular sport (32–34). Athletes, especially open-skill sports athletes, must rapidly adapt, inhibit pre-existing actions or responses, and change strategies (35, 36). Based on the findings that athletes have greater EFs than non-athletes, these EFs might not be easily improvable due to possible ceiling effects (37). In addition, athletes are highly physically active. As many studies show physical activity effectively supports EFs with respect to cognitive functions in different samples, such as healthy subjects (38), children (i.e., 6–13 years), and older adults (>50 years; review by (39), as well as patients with various diagnoses (40), the high physical activity could lead to the minimized effect of CT in athletes. On the other hand, meta-analytic evidence from studies investigating the effects of exercise on cognition demonstrates relatively similar effects across fitness levels (41), with the largest effects (*d* = 0.331) observed among individuals with a high fitness level. This evidence would suggest that despite high levels of physical activity, benefits for cognition may still be possible.

In addition, methodological issues have become apparent based on Harris's et al. (1) recent review. Even though Harris et al. (1) found training programs effective, especially when diagnostic tasks are similar to training tasks, evidence for CT improving performance on closely related tasks is sparse (17). Thus, the positive effects of a well-trained task might be due to the phenomenon of *teaching to the test* (42), meaning that the training task is too similar to the diagnostic task. Therefore, results showing improvements might have occurred due to training the same tested task rather than the concept under investigation (e.g., inhibition). The current study tries to avoid this methodological issue by providing a domain-generic training task that is only conceptually related to the diagnostic of EFs, especially inhibition. In other words, the athletes participating in this study practiced a domain-generic task (i.e., “Fruit Ninja”), and well-accepted computerized diagnostic tasks (i.e., Flanker task, number-letter task, n-back task) were used to test the changes in EF performance.

Overall, this current study aimed to examine the effects of a short-term (i.e., eight weeks) domain-generic CT (by smartphone app) intervention on EFs in young soccer players. Identifying whether a smartphone game might impact EFs relevant for in-game performance in a youth soccer player sample could favor the near transfer to soccer training. We hypothesized that this short-term domain-generic CT intervention using a smartphone app will lead to improvements in EFs. In detail, we assume that the effect is predominant for inhibition (Flanker task and Cued Go/NoGo task) as the tasks in “Fruit Ninja” have similarities with the Flanker task as they require inhibiting distractors (e.g., do not hit the “bombs” appearing on the screen). In addition, the game requires a Go or NoGo decision (Cued Go/NoGo task) because sometimes only distractors appear on the screen that are not to be touched (i.e., NoGo part). Further, we predicted the task to affect the young athletes' working memory by only a small degree because the task does not train the storage of information in working memory as, for example, a memory puzzle game would (22). Finally, we predicted that the intervention has relatively minor effects on cognitive flexibility as the task does not require different or switching strategies or task affordances.

## 2. Methods

### 2.1. Participants

An *a priori* statistical power analysis (F-test, MANOVA, repeated measures, within-between interaction) was conducted for sample size estimation based on the effect size of (43); *N* = 85, *η*2 = 0.17; Cohen's *f* = 0.47). Thus, with an alpha of 0.05 and a power of 0.80 for two groups, the sample size needed for the current study (calculated with G*Power 3.1) was *N* = 32.

**Figure 1:**
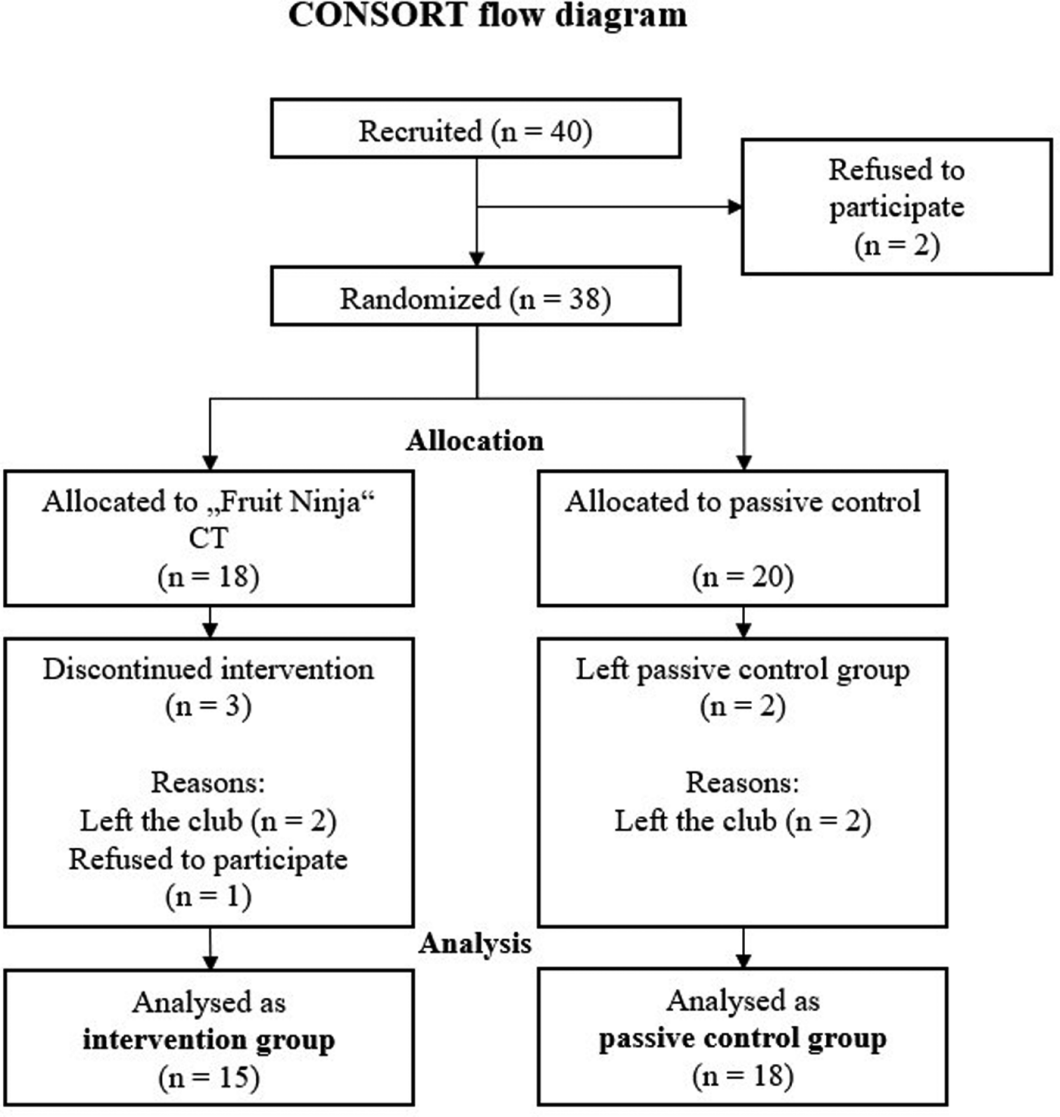
CONSORT flow chart.

**Figure 2:**
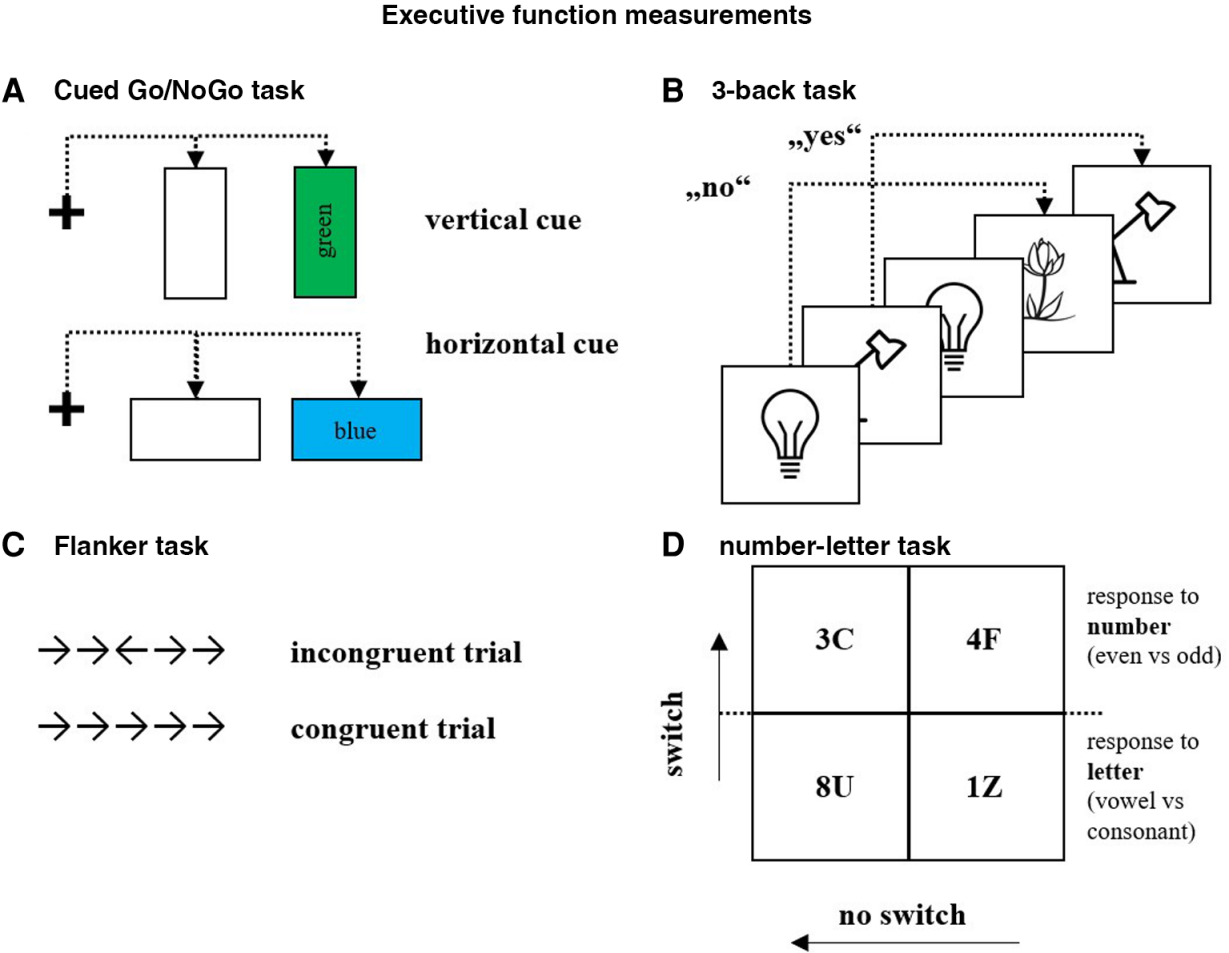
Executive function measurements: (A) Cued Go/NoGo task, (B) 3-back task, (C) Flanker task, (D) number-letter task.

Thirty-three youth soccer players aged 16–19 on average (mean = 17.03; SD = 0.94; first sample size was 40) volunteered to take part in this study (Figure 1). The players were from Germany's highest national league (Jugend A/B Bundesliga, U17-19). The players were randomly assigned to an intervention group (named IG; *n* = 15) or a passive control group (named CG; *n* = 18). The randomization was performed by Research Randomizer (https://www.randomizer.org/; 2 sets with 20 numbers describing the participants ID number; *n* = 40). Five players were excluded because they did not complete the intervention, as they either left the club (*n* = 4) or decided not to participate in the study (*n* = 1; see Figure 2). Five of the 15 in the IG and eight of the 18 players in the CG stated that they regularly play games on their computers or smartphones, showing no significant difference (χ^2^ = 0.424, *p* = 0.08, *V* = 0.03). The games played by the participants in the IG were exclusively sports games. Seven participants in the CG played sports games, one played ego shooter games, and two played role-playing games. The study protocol was in accordance with the ethical standards of the APA and the Declaration of Helsinki. The study was approved by the university's ethical committee (approval number of the ethical committee: 32/16). Informed consent was obtained from all the participants or their legal representatives. The authors declare that they comply with the Transparency and Openness Guidelines of the APA and will provide their data upon request. Age, body mass, body height, and gaming habits, did not significantly differ between groups (see Table 1).

**Table 1:** Anthropometric characteristics and gaming habits.

	Intervention group (IG) (*n* = 15)	Control group (CG) (*n* = 18)	*p*-value (by *t*-test)
Age (years)	16.8 (0.74)	17.22 (1.03)	0.209
Body mass (kg)	74.07 (7.45)	71.29 (6.61)	0.280
Height (m)	1.81 (0.07)	1.78 (0.06)	0.289
Training age (years)	5.00 (1.10)	5.11 (1.59)	0.826
Gaming time (min per day)	40.00 (37.81)	39.17 (59.40)	0.964

Data are mean (SD).

### 2.2. Measurements

The EFs were measured by computerized tasks using Inquisit Lab 6 (Millisecond Software LLC, Seattle, WA, USA) on a 17-inch screen and a QWERTZ keyboard.

#### 2.2.1. Inhibitory control

The Flanker task (44) and the Go/NoGo task (45) were used to assess inhibitory control.

For the Flanker task, the participants had to respond to a stimulus with five black arrows (four distracting arrows and the middle arrow as the target arrow) on a white background. For the congruent trials, all the arrows pointed in the same direction, and for the incongruent trials, the middle arrow pointed in the direction opposite to the distraction arrows (please see Figure 2A). In addition, the participants had to respond with the “I”-button if the target arrow pointed to the right and the “E”-button if it pointed to the left. The task consisted of four practice trials followed by 72 test trials (48 congruent and 24 incongruent; see (46); 4 min). The outcome values of the Flanker task were the response times for correct trials, as well as the accuracy of responses, in both congruent and incongruent conditions, mean response time of congruent and incongruent conditions, and total accuracy. Additionally, the flanker effect was calculated to describe the impact of the incongruency of stimuli difference in response times between the congruent and incongruent stimuli (45). The lower the flanker effect, the better inhibitory control of the participants.For the Cued Go/NoGo task (47), the participants were asked to press the spacebar when a stimulus with a green rectangle (Go) was displayed but refrain from pressing the spacebar when a blue rectangle was displayed (NoGo; Figure 2B). The blue and green rectangles could have been vertical or horizontal. The vertical rectangle had a high probability of being green (a Go trial), and the horizontal rectangle had a high probability of being blue (a NoGo trial). The participants received information about the orientation of the rectangle (the cue) shortly before the rectangle's color was revealed. Vertical cue Go/NoGo ratio was 4:1 (80% Go trials, 20% NoGo trials; higher probability of Go trials after vertical cue), and horizontal cue Go/NoGo ratio was 1:4 (20% Go trials, 80% NoGo trials; higher probability of NoGo trials after horizontal cue). The minimum number of trials required to fulfill the above conditions was 50. The task consisted of 250 trials, where each factor combination was repeated five times (10 min). The Go/NoGo task parameters were response times for the vertical and horizontal cued stimuli, and total error rate. Shorter response times and lower error rate indicate superior inhibition in the Go/NoGo task.

#### 2.2.2. Working memory

A computerized 3-back task (48) Figure 2C was used to assess the participants' working memory. The participants were presented with emotionally neutral pictures. They were asked to identify (by pressing the space bar) whether a picture had been presented to them three pictures back, thus, 3-back. The participants were given 23 test trials, followed by 46 target trials (4 min). The outcome values were response times for correct trials and accuracy for all responses. More correct responses (accuracy) and faster response times to correct stimuli represent better working memory.

#### 2.2.3. Cognitive flexibility

To assess cognitive flexibility, the number-letter task was modified from the Alternating-Runs-Switch task by (49). The participants were provided with a 2 × 2 matrix where a pair of a number and a letter appeared in one of the matrix fields (see Figure 2D). For the upper two boxes, the participants had to respond to the letter (consonant vs. vowel; 24 stimuli for practice, 32 regular stimuli) by pressing the “E”-button (on the left hand) for a consonant and the “I”-button (on the right hand) for a vowel. For the lower two boxes, the participants were required to respond to the digits by pressing as fast as possible (“I”- and “E”-button; odd vs. even; 24 stimuli for practice, 32 regular stimuli).

The participants were given 24 test trials in which only the letter or the number was presented, followed by 24 test trials where letters and numbers were both presented. Finally, 64 target trials, including 32 switch (switching from focusing on numbers to letters or vice versa) and 32 no-switch trials (continuing to focus on numbers or letters), were conducted (7 min). The response times and accuracy of responses for the number-letter task for the stimuli with and without a task switch were calculated. The so-called switch costs and the response time differences for the switch and no-switch trials were used as indicators of the participants' cognitive flexibility. Short response times, lower switch costs, and high accuracy are interpreted as indicators of superior cognitive flexibility.

### 2.3. Intervention

A smartphone game on a touch-based device was used for the CT intervention aimed improving EFs. Some evidence suggests that mobile respectively smartphone games, especially the application used in the study, could positively affect cognitive functions (for review, see 50). There is evidence that the use of the application “Fruit Ninja” in an immersive setting (VR) could enhance EFs (51). The goal of the game “Fruit Ninja” is to slice a variety of fruits appearing on the screen from different directions by mimicking a slashing gesture on the fruits. The players are awarded points for every fruit they slice and bonus points for combining slices and splitting multiple fruits. In addition, the players must ignore the bombs randomly appearing on the screen, and cutting through a bomb results in an instant loss in the game. As the game progresses, the number of stimuli (fruits and bombs) and the speed at which they appear progressively increase.

### 2.4. Procedures

The participants underwent pre-and post-test at their training facilities. The pre-test took place in September 2021, and the post-test was in November 2021 (the eighth week of the intervention period). First, the participants were instructed about the procedure and asked to sign the informed consent form. The participants were instructed not to consume caffeine or train for two hours before the testing. After that, they performed the cognitive tasks, which lasted approximately 45 min. The players were tested one hour before training to avoid physical exercise effects between 10:00 am and 4:00 pm. The tasks were randomly conducted in a quiet room. Finally, the experimenter explained the smartphone game to the participants. The participants were instructed to play the game five minutes per day and five days per week (working days). The participants of the control group were instructed to play the game or to change their gaming habits. The participants were not instructed to play on the weekends because no potential competition would have been affected. Additionally, the participants had to fill out a questionnaire so that their gaming behaviors at the post-test could be examined (the game time per day/week and the type of game, see Table 1).

### 2.5. Statistical analysis

The data were first checked for normal distribution and outliers. No outliers had to be excluded. Even though our data was not entirely normally distributed, parametric testing was performed, as an analysis of variance (ANOVA) is robust against violated normality test assumptions (e.g. 52). To examine whether inhibition (i.e., Go/NoGo; Flanker) changed over time due to the intervention, we ran a 2 (time: pre vs. post)×2 (group: IG vs. CG) multivariate analysis of variance (MANOVA) including response time parameters from the Go/NoGo task (i.e., response time for vertical cue trial; response time for horizontal cue trial; error rate) and an additional 2 (time: pre vs. post)×2 (group: IG vs. CG) MANOVA for parameters from the Flanker task (i.e., response time for incongruent trial). As accuracy parameters were not correlated to response time parameters, we ran two separate 2 (time: pre vs. post)×2 (group: IG vs. CG) ANOVAs for accuracy parameters for the Go/NoGo and Flanker tasks. For the effect of the intervention on working memory, two separate 2 (time: pre vs. post)×2 (group: IG vs. CG) ANOVAs were calculated separately for accuracy and response time, as accuracy and response time were not significantly correlated. To investigate the effect of the intervention on cognitive flexibility, we ran a 2 (time: pre vs. post)×2 (group: IG vs. CG) MANOVA, with accuracy and response time parameters as dependent variables. We then performed a posthoc analysis (univariate ANOVA and *t*-tests) using the Bonferroni correction method. The statistical analysis used SPSS 28 (SPSS, Chicago, IL, United States). A significance level of *p* < .05 was set.

## 3. Results

All descriptive statistics are presented in Table 2.

**Table 2:** Descriptive statistics of each variable.

Executive function	Task	Variable	Intervention group (IG)		Control group (CG)	
			Pre	Post	Pre	Post
Inhibition	Flanker task					
	Mean response time (ms)		440.3 ± 50.7	432.4 ± 54.8	403.1 ± 56.6	392.8 ± 50.7
	Response time, congruent (ms)		424.4 ± 48.0	419.2 ± 50.6	392.3 ± 53.9	384.3 ± 48.6
	Response time, incongruent (ms)		473.5 ± 60.2	459.7 ± 65.5	425.6 ± 67.6	410.4 ± 57.4
	Flanker effect (ms)		49.1 ± 28.3	40.5 ± 22.8	33.2 ± 32.9	26.1 ± 20.9
	Accuracy (%)		97.8 ± 1.2	98.0 ± 1.6	98.3 ± 1.5	98.3 ± 1.4
	Cued Go/NoGo task					
	Response time, vertical cue (ms)*		309.1 ± 17.9	299.8 ± 25.6	293.1 ± 23.9	308.2 ± 23.0
	Response time, horizontal cue (ms)		314.6 ± 19.9	308.2 ± 21.5	309.8 ± 20.9	321.8 ± 26.8
	Error rate (%)		0.6 ± 0.63	0.36 ± 1.05	0.91 ± 1.50	0.89 ± 1.20
Working memory	3-back task					
	Response time (ms)		811.6 ± 225.0	855.1 ± 269.3	858.3 ± 296.8	708.1 ± 173.3
	Accuracy (%)		14.6 ± 3.8	17.4 ± 4.8	15.3 ± 4.8	15.1 ± 6.7
Cognitive flexibility	Number-letter task					
	Response time, switch (ms)		1,382.5 ± 379.4	1,206.1 ± 345.1	1,495.5 ± 464.1	1,358.4 ± 290.8
	Response time, no switch (ms)		887.9 ± 148.7	804.5 ± 175.1	1,005.3 ± 224.5	866.1 ± 175.1
	Response time, switch cost (ms)		494.5 ± 285.4	401.5 ± 245.9	490.1 ± 343.4	492.2 ± 246.6
	Accuracy, switch (%)		89.0 ± 9.6	92.2 ± 6.9	90.2 ± 8.6	89.6 ± 9.1
	Accuracy, no switch (%)		95.7 ± 3.6	95.8 ± 5.0	97.6 ± 4.3	95.3 ± 5.8
	Accuracy, switch cost (%)		6.7 ± 7.5	3.6 ± 6.3	7.3 ± 6.6	5.6 ± 8.4

Data are mean ± SD.

* Significant (*p* < 0.05) time x group interaction.

### 3.1. Inhibition

For the reaction time parameters of the Cued Go/NoGo task, the results show a significant main interaction [*F*(3, 26) = 6.75, *p* = .015, *ηp*^2^ = 0.194]. However, the univariate testing confirm this effect for mean response time [*F*(1, 26) = 7.45, *p* = .011, *ηp*^2^ = 0.211]. This was followed up with two paired *t*-tests, and the results showed that the performance in the intervention group remained the same (*t*[14] = 1.08, *p* = .300, *d* = 0.30; *t*[14] = 1.13, *p* = .279, *d* = 0.30); however, the control group showed a significant decrease in mean response time and response time for vertical cued trials (*t*[16] = −2.63, *p* = .019, *d* = 0.65; *t*[16] = 2.82, *p* = .013, *d* = 0.70). No main effect was observed for time [*F*(3, 26) = 1.89, *p* = .156, *ηp*^2^ = 0.179]. We did not detect a significant effect for factor group [*F*(3, 26) = 0.12, *p* = .913, *ηp*^2^ = 0.00]. Error rate in the Cued Go/NoGo task did not show any main effect [*F*(1, 28) = 0.88, *p* = .357, *ηp*^2^ = 0.030] or interaction effects [*F*(1, 28) = 1.395, *p* = .247, *ηp*^2^ = 0.047].

For the Flanker tasks, the results for response times showed no main effect for time [*F*(3, 26) = 1.28, *p* = .295, *ηp*^2^ = 0.090] and no interaction effect [*F*(3, 26) = 0.33, *p* = .724, *ηp*^2^ = 0.025]. However, a significant main effect was detected for group [*F*(3, 26) = 2.892, *p* = .050, *ηp*^2^ = 0.258], showing that the control group was significantly better than the intervention group in response time measures (see Table 2). Accuracy in the Flanker task did not show any main effect (time: *F*[1, 27] = 0.01, *p* = .982, *ηp*^2^ < 0.01; group: *F*[1, 27] = 0.42, *p* = .522, *ηp*^2^ = 0.015) or interaction effects [*F*(1, 27) = 0.803, *p* = .378, *ηp*^2^ = 0.02].

### 3.2. Working memory

For the 3-back task, no effects were found for response times (time: *F*[1, 23] = 0.11, *p* = .748, *ηp*^2^ = 0.005; group: *F*[1, 23] = 1.21, *p* = .283, *ηp*^2^ = 0.05; interaction effects *F*[1, 23] = 2.36, p = .138, *ηp*^2^ = 0.09) or accuracy parameters (time: *F*[1, 23] = 1.54, *p* = .228, *ηp*^2^ = 0.06; group: *F*[1, 23] = 0.17, *p* = .688, *ηp*^2^ = 0.07; interaction effects: *F*[1, 23] = 2.60, *p* = .121, *ηp*^2^ = 0.102).

### 3.3. Cognitive flexibility

For the number-letter task, as a measure of cognitive flexibility, no main effect for group [*F*(4, 24) = 0.52, *p* = .725, *ηp*^2^ = 0.08] and no interaction effect [*F*(4, 24) = 1.85, *p* = .153, *ηp*^2^ = 0.235] were found. However, a significant main effect for time was found [*F*(4, 24) = 3.71, *p* = .017, *ηp*^2^ = 0.382]. The univariate testing confirmed this effect for response time in both switch trials [*F*(1, 24) = 8.55, *p* = .007, *ηp*^2^ = 0.204] and non-switch trials [*F*(1, 24) = 12.30, *p* = .002, *ηp*^2^ = 0.313]. The posthoc analyses showed that the overall participants in the IG showed faster response times in the switch trials [*t*(12) = 3.06, *p* = .010, *d* = 0.85], and the participants in both IG and CG showed faster response times in non-switch trials (IG: *t*[12] = 2.68, *p* = .020, *d* = 0.74; CG: *t*[12] = 2.47, *p* = .013, *d* = 0.62). In other words, the IG responded faster to both switch trials and non-switch trials from pre to post, and CG responded faster to non-switch trials from pre to post.

## 4. Discussion

This study aimed to examine the impact of an eight-week domain-generic CT intervention delivered through a smartphone app on EFs in young soccer players. The results revealed that the domain-generic CT intervention did not have a differential effect on EFs compared to a control condition without CT intervention. Specifically, no significant interactions were found for most of the dependent variables, except for the response time of the vertical cue in the Cued Go/NoGo task. Consequently, our hypothesis, which posited EF improvements following the smartphone app intervention (specifically, using Fruit Ninja), was not supported.

These findings are in contrast to prior research demonstrating a positive effect of domain-generic CT interventions on cognitive performance (22, 24, 25, 50). However, they are in line with several studies that also failed to show a positive effect of CT on EFs. In detail, results reported by Huang (51) and (53), which indicated either no effects of a game-based intervention or effects limited to a treatment group that engaged with the game in an immersive setting, such as virtual reality. Also, Oei and Patterson (54) found no improvement in EFs in undergraduate students who played “Fruit Ninja” for 20 h. Thus, it seems that playing more than triple as much as the athletes were asked to do in the current study also did not show an effect. One reason for the lack of effectiveness of the intervention can be the ceiling effect. Especially for inhibition, previous studies involving top-level athletes showed slightly higher response times for the Flanker task in comparison to the current (55). However, this interpretation should be drawn carefully, as different electronic devices can play a critical role in response time performance (56). Nevertheless, the participants in the current study were highly trained with generally expected high performance in non-sport-specific cognitive tasks, such as EF tasks (8, 35, 57). As our participants competed in the highest German league, it is plausible that they already had excellent EFs that could only be marginally improved. Additionally, for team sport athletes previous studies indicated that athletes from “strategic sports requiring adaptation to highly varying situations considering teammates, opponents, position, and objects” (55) or “open-skill sports” outperformed athletes from “closed-skill sports” in EF tasks (58, 59).

Furthermore, it can be argued that the lack of an effect of the intervention may be due to it being a domain-generic CT task. Previous studies have shown improvements in sport-specific tasks (1, 18), but this does not necessarily imply that the effects can be transferred to non-sport-specific or generic tasks. The findings of the meta-analysis by (60) indicate that the effects between high-skilled and low-skilled athletes are more evident when the stimuli and responses in cognitive tasks are specific. Moreover, (61) demonstrated that sport-specific perceptual-CT can lead to task-specific training effects in domain-specific tasks, but not necessarily transfer to perceptual-cognitive or soccer-specific performance.

The overall increase in cognitive flexibility is difficult to explain and might be an incidental finding. It could also be speculated that maturation set in during these eight weeks; however, this speculation is hard to justify. As two months seem to be a small range for maturation (62), we can also speculate that it is due to familiarization with the task. Even though this was not statistically significant, on a descriptive level, the athletes improved their performances in almost all EF parameters. In a previous study, we found that participants improve their EF performance by performing cognitive tasks (in a Soccerbot360) shortly after another, which can be attributed to learning effects (63). Whether this is the same for a time interval of eight weeks is unclear. If so, it needs to be determined in future research. However, this would present a problem for investigating the development of EFs and the effects of interventions on EFs. Thus, it might be useful in future studies to use tasks that assess the target concept (e.g., inhibition) but with different tasks in the pre- and post-test (e.g., Flanker task and Stroop task).

Several limitations should be considered when interpreting the results of this study. First, the participants' education level was not assessed. Factors such as academic outcomes, music education, and intelligence quotient have been shown to influence the expression of EFs (64, 62). Similarly, the participants' physical fitness (66) and emotional states, which have been found to be related to EF performance (63, 67, 68) were not assessed. Although efforts were made to control for these confounders, future studies are suggested to incorporate their assessment. Second, instead of randomization, matching groups by EF performance, as well as other demographic parameters, after the pre-test could have prevented unequal baseline for the intervention and control groups (IG vs. CG in pre-test: response time in vertical cued Go/NoGo task: *t*[30] = 2.11, *p* = .044, *d* = 0.75; response time in incongruent trial in Flanker task: *t*[29] = 2.13, *p* = .041, *d* = 0.75). In this study, the differences in baseline measure [e.g., the accuracy of the Number-letter task (no switch)] and the disordinal interaction effects [e.g., response time in Cued Go/NoGo task (vertical cue)] could limit the interpretability of intervention effects.Third, the progress of the intervention (i.e., high scores in the smartphone game) was not assessed. Differences in, or overall low motivation towards, playing the game might have resulted in a lack of effects of the intervention on EFs. Future studies should consider assessing performance in CT during the training intervention. Analyzing the participants' progress could help identifying the possible reasons for improvement or lack thereof in EFs due to CT. Finally, implementing a sedentary control group in the research design may help to understand the effects of expertise regarding the improvement of EFs.

## 5. Conclusions

The current research revealed no differences in EFs resulting from a smartphone-based CT task in high-level athletes. Therefore, the study's findings do not suggest an application of CT in the form of a smartphone-based game to improve EFs performance in soccer players. However, more research is needed to establish the efficacy of domain-specific interventions in high-level team sport athletes. Considering other confounding variables affecting EFs (e.g., emotional states and intelligence quotient) and monitoring the training process makes sense in terms of understanding the impact of CT on athletes of different levels. This might contribute to advanced knowledge in the field of sports performance-cognition interaction.

## Data Availability

The raw data supporting the conclusions of this article will be made available by the authors, without undue reservation.
